# Analysis of DNA strand-specific differential expression with high density tiling microarrays

**DOI:** 10.1186/1471-2105-11-136

**Published:** 2010-03-17

**Authors:** Luis Quintales, Mar Sánchez, Francisco Antequera

**Affiliations:** 1 Departamento de Informática y Automática, Facultad de Ciencias, Universidad de Salamanca, Pz. de Los Caídos s/n, 37007-Salamanca, Spain; 2 Instituto de Microbiología Bioquímica, Consejo Superior de Investigaciones Científicas (CSIC)/Universidad de Salamanca, Edificio Departamental, Campus Miguel de Unamuno, 37007-Salamanca, Spain

## Abstract

**Background:**

DNA microarray technology allows the analysis of genome structure and dynamics at genome-wide scale. Expression microarrays (EMA) contain probes for annotated open reading frames (ORF) and are widely used for the analysis of differential gene expression. By contrast, tiling microarrays (TMA) have a much higher probe density and provide unbiased genome-wide coverage. The purpose of this study was to develop a protocol to exploit the high resolution of TMAs for quantitative measurement of DNA strand-specific differential expression of annotated and non-annotated transcripts.

**Results:**

We extensively filtered probes present in Affymetrix Genechip Yeast Genome 2.0 expression and GeneChip *S. pombe *1.0FR tiling microarrays to generate custom Chip Description Files (CDF) in order to compare their efficiency. We experimentally tested the potential of our approach by measuring the differential expression of 4904 genes in the yeast *Schizosaccharomyces pombe *growing under conditions of oxidative stress. The results showed a Pearson correlation coefficient of 0.943 between both platforms, indicating that TMAs are as reliable as EMAs for quantitative expression analysis. A significant advantage of TMAs over EMAs is the possibility of detecting non-annotated transcripts generated only under specific physiological conditions. To take full advantage of this property, we have used a target-labelling protocol that preserves the original polarity of the transcripts and, therefore, allows the strand-specific differential expression of non-annotated transcripts to be determined. By using a segmentation algorithm prior to generating the corresponding custom CDFs, we identified and quantitatively measured the expression of 510 transcripts longer than 180 nucleotides and not overlapping previously annotated ORFs that were differentially expressed at least 2-fold under oxidative stress.

**Conclusions:**

We show that the information derived from TMA hybridization can be processed simultaneously for high-resolution qualitative and quantitative analysis of the differential expression of well-characterized genes and of previously non-annotated and antisense transcripts. The consistency of the performance of TMA, their genome-wide coverage and adaptability to updated genome annotations, and the possibility of measuring strand-specific differential expression makes them a tool of choice for the analysis of gene expression in any organism for which TMA platforms are available.

## Background

The introduction of gene expression DNA microarrays (EMAs) about 15 years ago opened a whole new range of possibilities for studying genome dynamics by making possible the simultaneous analysis of the transcription of all the genes in a genome [1]. Genes are represented in EMAs either by a reduced number of oligonucleotides (around 11) or by PCR-synthesized fragments spanning a fraction of their length. The advent of genomic tiling microarrays (TMAs) expanded the possibilities of EMAs by increasing the number of probes so that complete genome coverage could be reached. TMAs are widely used for structural and functional genome analyses, which include the localization of protein-DNA interactions by chromatin immunoprecipitation followed by microarray hybridization (ChIP on chip), the mapping of DNA methylation and histone modifications, nucleosome positioning, DNase hypersensitive regions and the assessment of copy number variation, among other applications (reviewed in [2]).

The generation of high-resolution transcription maps by hybridizing total RNA to TMAs has uncovered the existence of a large variety of RNAs, many of which are non-coding, in a range of organisms that include *Bacillus subtilis *[3], *Saccharomyces cerevisiae *[4], *Schizosaccharomyces pombe *[5], *Caenorhabditis elegans *[6], *Drosophila *[7], human [8] and *Arabidopsis *[9]. This unprecedented view of the transcriptional landscape of the genome derives mainly from a qualitative interpretation of TMA analysis, and raises the challenge of establishing the putative biological role of non-annotated transcriptionally active regions. A step towards assigning functions to these transcripts is their quantitative analysis to facilitate comparisons between different physiological conditions. In principle, the much higher density of the probes of TMAs and the possibility of providing unbiased information about transcription directionality and antisense transcription should offer several advantages over EMAs for measuring differential gene expression. One disadvantage in the use of TMAs for expression analyses, however, is the requirement of more sophisticated bioinformatic tools to process the hybridization signal from several million probes that have not been classified as genic or intergenic. In contrast, the number of probes in EMAs is at least one order of magnitude lower; they are unambiguously ascribed to specific genes, and the processing and summarization of their hybridization signal is relatively straightforward.

Here we report a probe-filtering protocol to generate custom Chip Description Files (CDF) to process the hybridization signals of TMAs from each DNA strand in a quantitative manner to measure differential transcriptional expression. CDFs can be generated from any genome annotation or any set of probes in a microarray and they allow direct use with the same tools as those used for the analysis of differential expression with EMAs. We experimentally compared the performance of the Affymetrix TMA and EMA platforms hybridized with identical RNA samples from the yeast *Schizosaccharomyces pombe *to measure differential gene expression under conditions of oxidative stress. We also compared our results with those from a previous study using custom-made microarrays based on PCR amplified probes representing over 4500 *S. pombe *genes [10,11]. Our results show that TMAs are as reliable as EMAs for measuring the differential expression of protein coding genes. In addition, by combining the high resolution of TMAs with a labelling protocol that preserves the polarity of RNA, we show that they allow the quantitative analysis of previously unidentified strand-specific non-annotated and sense/antisense transcripts.

## Methods

### *Schizosaccharomyces pombe *culture growth, oxidative stress conditions, and RNA isolation

Cultures of *S. pombe *wild-type strain 972 h- were grown under identical conditions to those described by Chen et al. [11] in 100 ml yeast extract (YE) medium at 30°C and 170 rpm up to OD_595_ = 0.2 (4 * 10^6 ^cells/ml). Two separate cultures developed from independent single colonies were processed in parallel throughout the entire experiment (biological duplicates). Cells from a 30 ml volume were collected by centrifugation at 2000 rpm for 2 minutes and the pellet was immediately frozen in liquid nitrogen. Hydrogen peroxide (SIGMA, H-1009) was added to the rest of the culture at a final concentration of 0.5 mM and incubation was allowed to proceed for 30 minutes, after which 30 ml of culture were processed as above.

Total RNA was prepared by resuspending the cell pellets in 20 *μ*l extraction buffer (100 mM EDTA, pH 8.0, 100 mM NaCl, 50 mM Tris-HCl, pH 8.0), 20 *μ*l phenol/chloroform, 2 *μ*l 10% SDS, 200 *μ*l glass beads (425-600 *μ*m, SIGMA G-8772). Cells were mechanically disrupted in a Fast-Prep device (Savant BIO 101) and the cell lysate was extracted with phenol, phenol/chloroform and chloroform/isoamyl alcohol before precipitation with 0.3 M sodium acetate and ethanol. RNA was resuspended in 50 *μ*l of sterile water with diethyl pyrocarbonate (SIGMA D-5758) and was further purified with the RNeasy mini kit (Quiagen) following the supplier's specifications.

### Target labelling and microarray hybridization

To hybridize the Affymetrix Genechip Yeast Genome 2.0 expression microarrays (EMA), 7 *μ*g of total RNA was used for cDNA synthesis. Target labelling was performed following the instructions of the Affymetrix GeneChip whole transcript double-stranded target-labelling assay manual. To hybridize the Affymetrix GeneChip *S. pombe *1.0FR tiling microarray (TMA), 300 ng of total RNA without rRNA reduction was used for cDNA synthesis. Target labelling preserving the original polarity of RNAs was performed following the instructions of the GeneChip whole transcript sense target labelling assay manual from Affymetrix. Biological duplicates from cells treated and not treated with 0.5 mM hydrogen peroxide were used to hybridize TMAs and EMAs. The Pearson correlation coefficients of the probe hybridization signals between TMA duplicates hybridized with RNA from untreated and hydrogen peroxide-treated samples were 0.997 and 0.996, respectively. In the case of EMAs, the Pearson correlation coefficients were 0.998 and 0.998, indicating minimum variability between duplicates. The complete set of microarray hybridization results is available at the GEO database under accession number GSE19020.

### Differential expression analyses

For differential expression analyses, microarray probe intensities were processed using the Robust Multiarray Average (RMA) procedure, which includes RMA background adjustment, quantile normalization, and median polish summarization [12].

### Segmentation algorithm for non-annotated differentially transcribed regions (dTRs)

The segmentation algorithm used to define the boundaries of non-annotated differentially transcribed regions (dTRs) included only probes displaying a difference in the hybridization signal above 0.8 (log2 scale). Probes less than 60 nucleotides apart (approximately 3 tiling probes) were clustered in a single region. Only regions larger than 180 nucleotides with an average hybridization signal difference of all the probes included above 0.8 (log2 scale) were selected. Regions meeting these criteria were fused if the distance between them was shorter than 120 nucleotides.

## Results and Discussion

### Generation of custom Chip Description Files (CDF) for expression analyses

**Figure 1:**
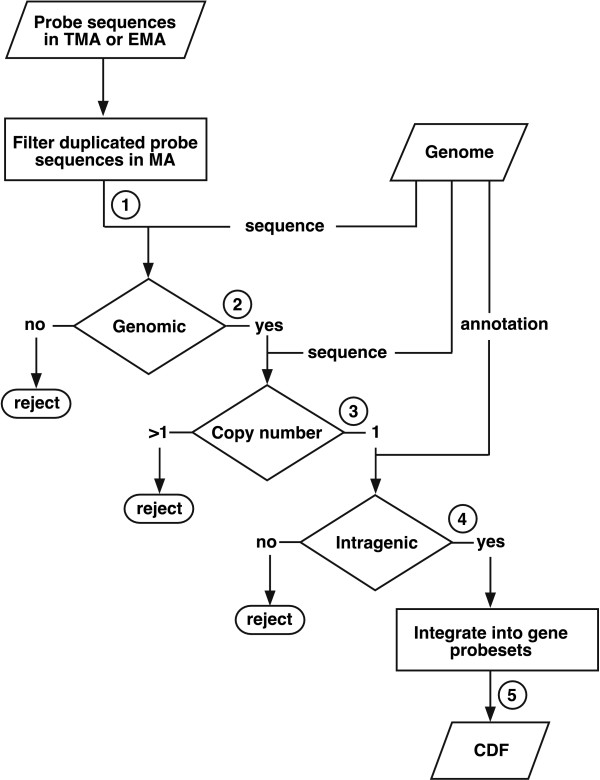
**Flowchart of custom CDF construction**. The five steps involved in the filtering and selection of probes present in TMAs or EMAs to integrate them into probesets and generate custom CDFs are indicated. See text and Table 1 for details.

Analysis of differential gene expression using microarrays requires the generation of a Chip Description File (CDF), which links each position in the microarray to a specific gene. For the specific analyses addressed in this work, we generated several custom CDFs following the steps indicated in the flowchart shown in Figure 1.

In the first step, the sequences of probes in the Affymetrix platforms are filtered so that no more that one probe with the same nucleotide sequence will be present in the CDF. In a second step, each single copy probe is mapped against the complete genome sequence used as a reference and those not matching are discarded (this can result from sequence updating after the microarray was designed). This process requires intensive computation since more than a million probes (Table 1) must be mapped onto the 14.1 Mb of the *S. pombe *genome (in which the 1.2 Mb of the rDNA locus was excluded). This step was optimized using a Karp-Rabin algorithm [13], adapted to a four-size alphabet. The marked decrease from 120432 to 54022 probes in the EMA platform (Sp_EMA columm in Table 1) was mostly due to the removal of probes against the *Saccharomyces cerevisiae *genome included in the Affymetrix Genechip Yeast Genome 2.0 expression microarrays. In a third stage, probes mapping to more than one position in the genome are also discarded. In the fourth step, each probe is mapped against the corresponding genome annotation to select only those matching exons or predicted open reading frames (ORF). As a reference, we used the Sanger Centre genome annotation release of July 17, 2009, which includes 5063 genes. The fifth step integrates all remaining probes into probesets that represent the number of ORFs in a given genome annotation. Genes represented by less than four probes in TMAs or EMAs were not incorporated into the final CDFs because this has been reported to be the lowest number of probes able to provide statistically significant results [14]. The CDF files generated can be used directly with current tools for the differential expression analysis of EMAs, such as the Robust Multichip Analysis (RMA) method [12].

**Table 1:** Number of probes and probesets during generation of CDFs

	Sp_TMA		Sp_EMA		Sp_PCR_TMA		Sp_PCR_EMA
Total probes in MA	1174792		120855				

Unique probes in MA (Step 1)	1145245		120432				

Probes in Genome (Step 2)	1130689		54022				

Unique probes in Genome (Step 3)	1108696		53232				

Intragenic probes (Step 4)	316114		52756		74418		

Probe sets (genes) in CDF (Step 5)	4972		4904		4574		4574

Following this scheme, we generated three custom CDFs (Table 1):

1. CDF "Sp_TMA". This included probes from the Affymetrix GeneChip *S. pombe *1.0FR tiling array filtered as described above to generate 4972 probesets.

2. CDF "Sp_EMA". This included probes from Affymetrix GeneChip Yeast Genome 2.0 filtered to generate 4904 probesets. We used the same genome annotation as in the Sp_TMA CDF to make the results comparable between both platforms.

3. CDF "Sp_PCR_TMA". To compare results from both Affymetrix platforms and custom designed microarrays developed in the Sanger Centre [10], as a reference in step 4 (Figure 1) we used the sequence of the amplicons used as probes in the Sanger microarray. As a result, 4574 probesets were generated from the Affymetrix TMA 1.0FR matching sequences in the Sanger amplicons. We have called the original Sanger custom microarray "Sp_PCR_EMA".

The Perl software used and the custom CDFs generated for expression analysis can be downloaded from our web site http://genomics.usal.es/TMADE.

### Probe density and number of genes analyzed using different platforms

**Figure 2:**
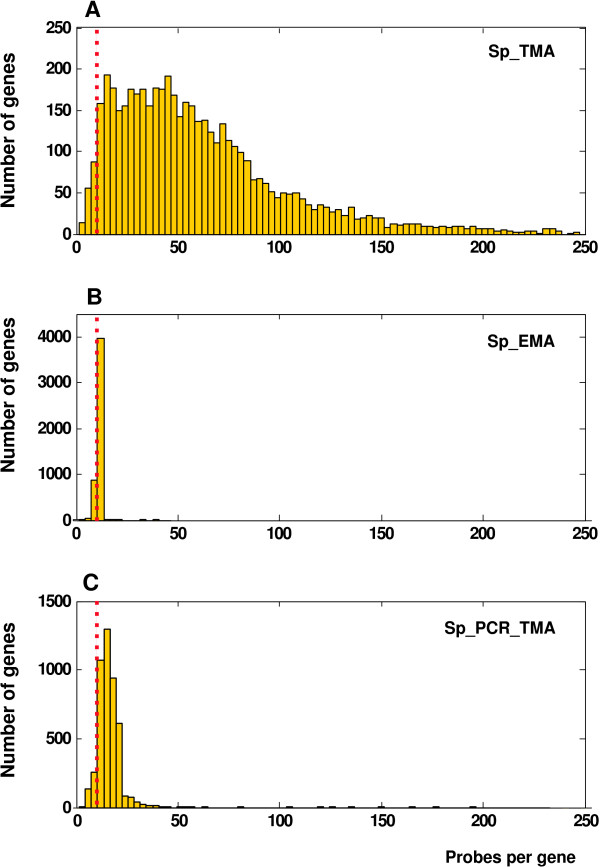
**Frequency of probes per gene in custom CDFs**. The dashed red line indicates the 11-nucleotide boundary. A) 96.8% of all *S. pombe *genes are represented by more than 10 probes in Sp_TMA CDF. B) 11.7% and 80.9% of all genes are represented by 10 or 11 probes, respectively, in Sp_EMA CDF. C) 91.5% of all genes are represented by more than 10 probes in Sp_PCR_TMA CDF.

As shown in Table 1, the total number of probes matching genes (Step 4) is 6-fold higher in Sp_TMA than in Sp_EMA because the number of probes per gene is proportional to their size in Sp_TMA. This means that 96.8% of all the genes are represented by 11 or more probes in Sp_TMA, while 11.7% and 80.9% are represented by 10 or 11 probes in Sp_EMA CDF, respectively (Figure 2A and 2B). In addition, Sp_TMA allowed the analysis of 68 genes and 398 genes not present in Sp_EMA or Sp_PCR_EMA, respectively. Because the amplicons in Sp_PCR_EMA range between 180-500 bp, 91.5% of the genes are represented by 11 or more probes in Sp_PCR_TMA (Figure 2C).

**Figure 3:**
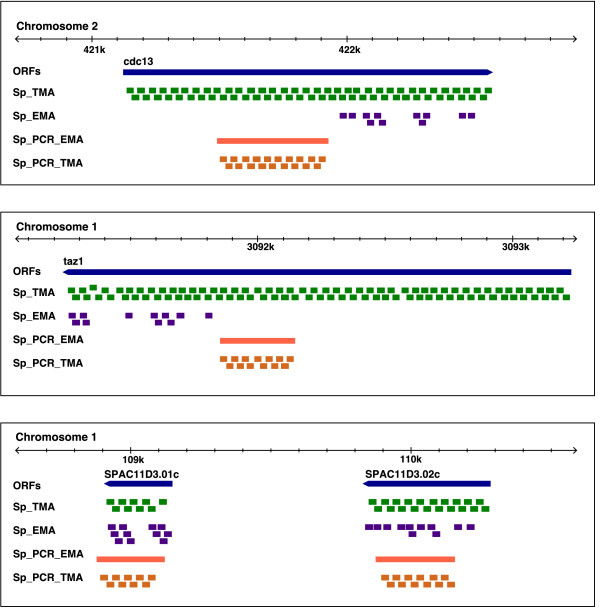
**Probe coverage of genes in different microarray platforms**. ORFs of the *cdc13*, *taz1*, *SPAC11D3.01c *and *SPAC11D3.02c *genes are indicated by blue bars with pointed ends towards the direction of transcription. Green and purple rectangles represent probes covering the four ORFs in the Sp_TMA and Sp_EMA CDFs, respectively. Orange bars indicate PCR-amplified fragments in the Sp_PCR_EMA microarray designed by Lyne et al. [10], and Sp_PCR_TMA orange rectangles represent probes in Sp_TMA corresponding to PCR fragments in Sp_PCR_EMA. The size of the three genomic regions shown is 2.2 kb. Genomic coordinates are indicated at the top of each panel. Genome-wide probe coverage in the four different custom CDFs can be accessed from the genome browser on our website http://genomics.usal.es/TMADE/browser.

The differences in the probe coverage of specific genes in the different platforms are illustrated in Figure 3. ORFs of average size, such as *cdc13 *(1449 bp) or *taz1 *(1992 bp), are represented by 67 and 92 probes, respectively, in Sp_TMA, and by 11 in both cases in Sp_EMA. Only very small genes, such as *SPAC11D3.01c *(240 bp), would be represented by a lower number of probes in Sp_TMA than in Sp_EMA. This disadvantage would affect 82 annotated ORFs shorter than 240 bp, which represent only 1.6% of all *S. pombe *genes. Figure 3 also shows the amplicons representing these four ORFs in Sp_PCR_EMA and the corresponding probe coverage in Sp_PCR_TMA.

### Comparative analysis of differential gene expression

**Figure 4:**
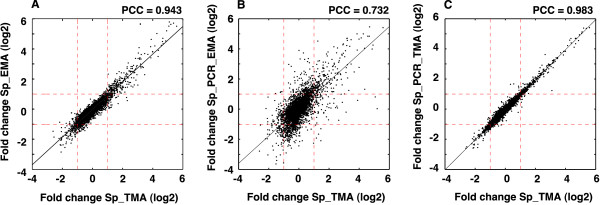
**Comparative analyses of differential gene expression using different platforms**. A) Correlation between the level of differential gene expression (log2) of *S. pombe *genes under conditions of oxidative stress in the Sp_TMA and Sp_EMA platforms. B) Correlation between Sp_TMA and Sp_PCR_EMA platforms. C) Correlation between Sp_TMA and the fraction of probes in the Affymetrix 1.0FR microarray matching sequences in the PCR-amplified fragments that make up the Sp_PCR_EMA microarray. Dashed red lines indicate a two-fold level of positive or negative differential expression as detected by each platform. Pearson correlation coefficients (PCC) are indicated at the top of each panel.

We undertook a comparative analysis of differential gene expression of *S. pombe *under conditions of oxidative stress using the TMA and EMA platforms. Figure 4A shows that the Pearson correlation coefficient between the levels of differential expression of the 4904 genes detected by Sp_EMA and their corresponding counterparts in Sp_TMA is 0.943, indicating the similar performance of both platforms. The coefficient, however, dropped to 0.732 upon comparing Sp_TMA and Sp_PCR_EMA (Figure 4B). This lower correlation could be due to the use of different microarray platforms and hybridization conditions [11] or to the localization of probes relative to genes (Figure 3). To distinguish between these possibilities, we generated a Sp_PCR_TMA CDF as described above and compared the differential expression detected in both cases. The correlation coefficient rose to 0.983 (Figure 4C), which was even better than that seen in Figure 4A, probably because the average number of probes per gene is higher in Sp_PCR_TMA than in Sp_EMA. This implies that differences between Sp_TMA and PCR-based Sp_PCR_EMA are likely due to the use of different platforms, labelling protocols, and processing of the results, as has also been reported in other comparative studies [15]. The numerical values of differential expression between the platforms on which the correlations in Figure 4 were calculated are shown in Additional File 1.

Taken together, these results show that the Sp_TMA and Sp_EMA Affymetrix platforms yielded virtually identical results, thus validating the use of TMAs for the analysis of differential expression of annotated genes. These results are consistent with those reported in a previous study carried out in *Arabidopsis*, in which a strong correlation between the performance of EMAs and TMAs for quantitative gene expression was also found [9]. The fact that the correlation between the results from both platforms was higher in our study could be due to a more precise annotation of the *S. pombe *genome relative to *Arabidopsis *or to the fact that repetitive probes were not filtered out in that study.

### Quantitative analysis of DNA strand-specific transcription and of non-annotated transcripts

**Figure 5:**
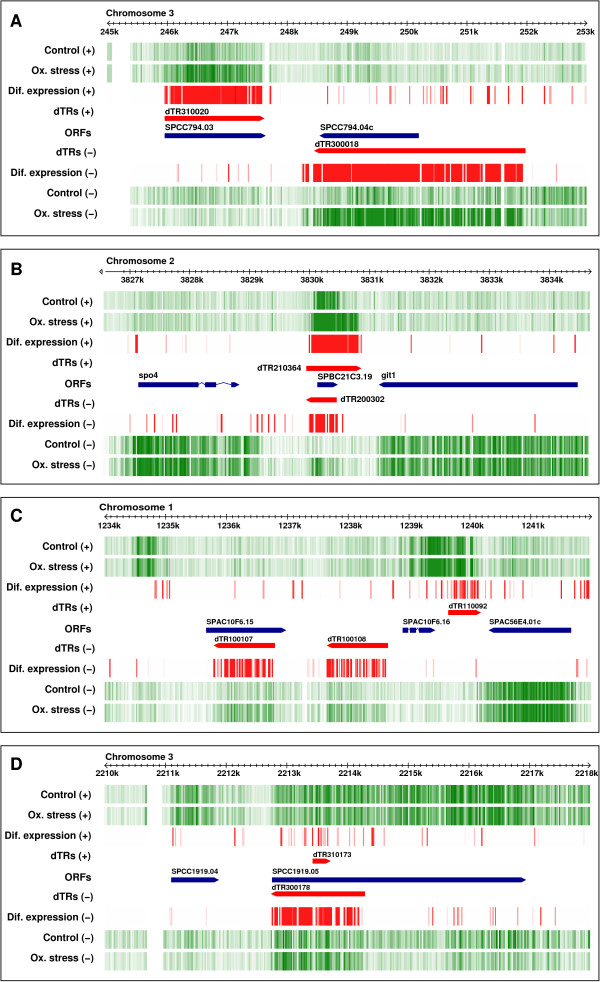
**Browser visualization of differential expression**. Vertical green lines represent transcription from both DNA strands (indicated by + or -) in control *S. pombe *cells and in cells under oxidative stress. Darker green indicates a higher level of expression. Red vertical lines indicate a differential over- or under-expression level between both physiological conditions greater than 1.75-fold. Differences below this level are not shown. ORFs are indicated by blue bars pointing towards the direction of transcription. Differentially transcribed regions (dTRs) showing differential expression are represented by red bars. A) Overexpression of dTR310020 and dTR300018 from complementary strands encompassing the *SPCC794.03 *and *SPCC794.04c *genes under oxidative stress. B) Overexpression of dTR210364 and antisense dTR200302 from complementary strands encompassing the *SPBC21C3.19 *gene. C) Overexpression of non-annotated dTR100108 and dTR110092. Antisense transcription is also detected across *SPAC10F6.15 *under conditions of oxidative stress. D) Strong overexpression of antisense RNA (dTR300178) from a 1.5 kb region at the 5' end of the *SPCC1919.05 *gene. Quantitative data for all differentially expressed annotated and non-annotated dTRs across the complete genome are shown in Additional File 2. The data can be accessed from the genome browser on our website http://genomics.usal.es/TMADE/browser.

The use of a labelling protocol that preserves the polarity of transcribed RNA allowed the detection of differentially transcribed regions (dTRs) from each DNA strand (Figure 5A). This experimental approach is so sensitive that it can measure differential expression even between sense and antisense transcription from the same genomic region, as in the case of the *SPBC21C3.19 *gene (Figure 5B). Two examples of antisense dTRs (dTR100107 and dTR300178) are shown in Figures 5C and 5D). One of the main advantages of TMAs over EMAs is the possibility of identifying non-annotated dTRs. Measurement of their differential expression requires the genomic coordinates of their boundaries to be established prior to the generation of custom CDFs. To this end, we used a segmentation algorithm based on the log2 ratio of the probe-by-probe differential hybridization signal after quantile normalization of the signal for each independent hybridization experiment. This strategy does not require previous generation of a transcriptome map since it selects only differentially expressed probes across the genome regardless of whether they map to previously identified ORFs, and it excludes the large majority of genes and non-annotated transcripts whose expression is not affected under the experimental conditions tested. For segmentation, we used a sliding-window strategy coupled to a thresholding criterion [16], as described in Materials and Methods. Once the boundaries of the dTRs had been established, they were used as input to generate a custom CDF, following the strategy described in Figure 1 for quantitative analysis. By using this approach, we detected 1546 dTRs showing a higher than two-fold differential expression, of which 510 (33.0%) did not overlap annotated ORFs, such as dTR100108 and dTR110092 (Figure 5C). The quantitative data for differentially expressed non-annotated dTRs across the entire *S. pombe *genome are shown in Additional File 2 and can be visualized on the genome browser of our web site http://genomics.usal.es/TMADE/browser.

The development of DNA microarrays and more recently of deep sequencing technologies has revealed that in addition to protein coding genes, a large fraction of eukaryotic genomes are transcribed. Detailed transcriptome maps in *Saccharomyces cerevisiae *have uncovered an unexpectedly large amount of stable and unstable non-coding RNAs, a large fraction of which are transcribed bidirectionally from nucleosome-free regions [17,18]. In order to assess the biological role of these trancripts, the approach described here should be useful to measure their differential expression under different physiological conditions. It could also be adapted to the analysis of the allele-specific expression that has been recently reported in *S. cerevisiae *[19]. The possibility of assigning polarity to non-annotated dTRs is essential for predicting possible RNA secondary structures that could be relevant to their function. This is particularly well illustrated by the human HARF1 non-coding transcript, which derives from one of the most divergent regions between humans and chimpanzees [20] and is one out of several candidate genes that could contribute to establishing differences between both species.

## Conclusions

We have shown that information derived from TMA hybridization can be simultaneously processed for high-resolution qualitative and quantitative analysis of differentially transcribed regions. The consistency of the performance of TMAs, their genome-wide coverage, and their adaptability to updated genome annotations, together with the possibility of quantitative measurement of the differential expression of non-annotated and antisense transcripts, makes them a tool of choice for the analysis of genome dynamics in any organism for which TMA platforms are available.

## Authors' contributions

LQ designed the computational methods and implemented the algorithms. MS performed the biological assays and prepared RNA samples for microarray hybridization. FA designed and supervised the general strategy of the work. LQ and FA wrote the article. All authors analyzed data, discussed results and approved the final version.

## Supplementary Material

Additional file 1 **Differential expression of annotated ORFs in *Schizosaccharomyces pombe *growing under oxidative stress**. Columns indicate the following: (A) Gene name. Different synonyms for each gene are indicated, (B) number of exons, (C) ORF length, (D) number of probes per probeset in Sp_TMA, (E) Differential expression (log2) in stressed relative to non-stressed cells in Sp_TMA (orange), (F) Corresponding p-value, (G) number of probes per probeset in Sp_EMA, (H) Differential expression (log2) in Sp_EMA (green), (I) corresponding p-value, (J) Differential expression (log2) in Sp_PCR_EMA (blue), (K) number of probes in PCR amplicons used as probes in Sp_PCR_TMA, (L) Differential expression (log2) in Sp_PCR_TMA (purple), (M) corresponding p-value. Click here for file

Additional file 2 **Differentially transcribed regions (dTRs) in *Schizosaccharomyces pombe *under oxidative stress**. Columns indicate the following: (A) identification number assigned to each dTR under the experimental conditions used, (B) differential expression (log2) in stressed relative to non-stressed cells, (C) corresponding p-value, (D, E) chromosome number, strand polarity and genomic coordinates of dTR initiation and end, (F) dTR length, (G) Empty cells indicate no overlap between dTR and any annotated transcript in the Sanger Centre genome release of July 17, 2009. Different synonyms for each gene are indicated. A symbol < or > preceding the name of the gene indicates that one end of the annotated ORF maps within the dTR and the other lies beyond its starting or end boundary, respectively. Symbols > < flanking the name of a gene indicate that the entire ORF is included within the boundaries of the dTR. Symbols < > flanking the name of the gene indicate that the entire dTR is included within the boundaries of the ORF. The 2328 entries in the table correspond to 2124 dTRs (Column A). The difference is due to the fact that some dTRs overlap with several exons. Out of the 2328 entries, 1546 were differentially expressed at least 2-fold and were distributed as follows: 510 dTRs (33.0%) had an average length of 613 nucleotides and did not overlap with annotated ORFs; 346(22.4%) were entirely included within ORFs and the remaining 690 (44.6%) overlapped partially with annotated ORFs. These data can be accessed from the genome browser on our website http://genomics.usal.es/TMADE/browser. Click here for file
